# The Importance of Sleep in Overcoming Childhood Obesity and Reshaping Epigenetics

**DOI:** 10.3390/biomedicines12061334

**Published:** 2024-06-15

**Authors:** Erika Richter, Priyadarshni Patel, Jeganathan Ramesh Babu, Xu Wang, Thangiah Geetha

**Affiliations:** 1 Department of Nutritional Sciences, Auburn University, Auburn, AL 36849, USA; 2 Boshell Metabolic Diseases and Diabetes Program, Auburn University, Auburn, AL 36849, USA; 3 Alabama Agricultural Experiment Station, Auburn University, Auburn, AL 36849, USA; 4 Department of Pathobiology, College of Veterinary Medicine, Auburn University, Auburn, AL 36849, USA; 5 HudsonAlpha Institute for Biotechnology, Huntsville, AL 35806, USA

**Keywords:** epigenetics, childhood obesity, sleep, DNA methylation, histone modifications, non-coding RNAs

## Abstract

The development of childhood obesity is a complex process influenced by a combination of genetic predisposition and environmental factors, such as sleep, diet, physical activity, and socioeconomic status. Long-term solutions for decreasing the risk of childhood obesity remain elusive, despite significant advancements in promoting health and well-being in school and at home. Challenges persist in areas such as adherence to interventions, addressing underlying social determinants, and individual differences in response to treatment. Over the last decade, there has been significant progress in epigenetics, along with increased curiosity in gaining insights into how sleep and lifestyle decisions impact an individual’s health. Epigenetic modifications affect the expression of genes without causing changes to the fundamental DNA sequence. In recent years, numerous research studies have explored the correlation between sleep and the epigenome, giving a better understanding of DNA methylation, histone modification, and non-coding RNAs. Although significant findings have been made about the influence of sleep on epigenetics, a notable gap exists in the literature concerning sleep-related genes specifically associated with childhood obesity. Consequently, it is crucial to delve deeper into this area to enhance our understanding. Therefore, this review primarily focuses on the connection between sleep patterns and epigenetic modifications in genes related to childhood obesity. Exploring the interplay between sleep, epigenetics, and childhood obesity can potentially contribute to improved overall health outcomes. This comprehensive review encompasses studies focusing on sleep-related genes linked to obesity.

## 1. Introduction

Obesity represents a worldwide health issue associated with a range of conditions and ailments, including type 2 diabetes, high blood pressure, heart disease, stroke, and cancer [1]. The development of obesity is a complex process, influenced by multiple interactions among behavioral, environmental, and genetic factors [1]. According to the latest report from the World Health Organization (WHO), there are more than 1.9 billion adults grappling with excess weight, with over 650 million falling into the obese category [2]. Collectively, this accounts for approximately 13% of the global adult population; however, it does not stop there [2]. The escalating prevalence of childhood obesity is even more concerning, affecting millions of children worldwide [3]. According to WHO’s database, an estimated 39 million children were overweight or obese in 2020 [2]. Obesity is a multifaceted condition marked by the accumulation of excess or unhealthy body fat, carrying a multitude of health implications [1]. Today, the shift towards a more sedentary way of life, coupled with higher dietary fat and sugar consumption, may be linked to this significant surge in obesity [4]. As preventative medicine becomes more readily available, it is imperative to further understand the various factors that play a major role in the development of childhood obesity. In recent years, there have been significant advancements in the field of epigenetics [3]. Epigenetics is the study of how external factors influence the way genes operate, without altering the DNA sequence itself [5]. These epigenetic markers modify the local chromatin environment, affecting DNA accessibility and the regulation of DNA-dependent mechanisms, such as gene transcription [6,7]. External factors, such as diet, amount of sleep and physical activity, and exposure to pollutants, contaminants, and pesticides, can affect epigenetic marks [8,9,10,11,12]. This, in turn, can lead to changes in gene expression patterns, with significant implications for overall health and well-being [8]. 

The innate need for sleep is fundamental to the maintenance of optimal health. Studies have shown that sufficient sleep is an essential component for upholding a healthy way of life; nonetheless, countless people across the globe do not achieve enough of it [13]. As in adults, inadequate sleep in children can have a negative impact on physical and mental health, as well as on cognitive performance, thus translating to limited performance in school and extracurriculars [14]. Furthermore, an adequate night of sleep has been known to have a significant effect on long-term health as well, helping to reduce the risk of diseases such as obesity, diabetes, cancer, and heart disease, as well as promoting growth and longevity [8,15,16]. Despite the lack of sleep being a large societal burden, it remains under-recognized as a significant measurement of health [17]. Epigenetic changes can occur in response to environmental factors, one of the most important being sleep [8]. Obesity is linked to chronic sleep deprivation, which has been identified as a potential risk factor in today’s industrialized society, affecting around 1 in 3 adult Americans [18,19]. Optimal sleep encompasses various dimensions, such as sufficient duration, suitable timing, and high quality [20]. When these characteristics of adequate sleep are not met, the potential for negative outcomes increases [20]. Research has demonstrated that disrupted sleep patterns can impact epigenetic makeup in a multitude of ways, including the direct inhibition of enzymes responsible for DNA methylation or histone modifications, or altering the availability of pivotal substrates required for specific enzymatic processes [8]. Consequently, understanding the intricate relationship between sleep and the epigenome, and how this, in turn, influences gene expression, is imperative for the advancement of enhanced sleep therapies and educational efforts aimed at preventing childhood obesity and managing various metabolic disorders [8]. Therefore, understanding the impact of sleep on the epigenome and its subsequent influence on gene expression is vital for enhancing public awareness regarding the significance of sleep as a preventive measure or therapeutic approach for various metabolic diseases.

There is substantial evidence demonstrating how consistent insufficient sleep has been linked to negative health consequences, such as obesity, type 2 diabetes mellitus (T2DM), cardiovascular problems, and overall mortality [17,21,22,23,24,25,26]. Inadequate sleep is also connected to daytime drowsiness, tiredness, a lowered mood, and overall reduced daytime functioning [27]. When considering these characteristics in relation to childhood obesity, the results suggest that the negative consequences of insufficient sleep contribute to the rising rates of adiposity in youth due to several external factors [27]. Most of these behavioral and environmental factors stem from today’s increased sedentary lifestyle due to increased screen time, a decreased amount of physical activity both at home and at school, and the overall repercussions of COVID society. These factors that society faces today all contribute to the rise of childhood obesity; however, evidence-based research has now shifted its focus to what is occurring at the epigenetic level in relation to the duration and quality of sleep and overall health, thus emphasizing the importance of sleep as a fundamental health determinant [28,29]. Several cross-sectional and longitudinal epidemiological studies, both of children and adults and across different ancestries and geographical environments, suggest that both short and long sleep durations are risk factors for obesity, and consequently, many other metabolic disease states [21,22,23,24,30,31]. The epidemiological data suggest a stronger link between sleep duration and obesity in children compared to adults, with the effect size diminishing with age [32]. However, there are a limited number of studies exploring sleep aspects beyond duration and focusing on how obesity affects sleep rather than investigating the influence of sleep on obesity from an epigenetic standpoint [32]. Therefore, it is imperative to look further into the epigenetic landscape of sleep and its connection to obesity [33].

While there have been profound discoveries regarding the impact of sleep on epigenetics, there is currently a gap in the literature regarding epigenetic mechanisms of sleep-related genes, specifically concerning childhood obesity. Therefore, gaining further insight on this topic is imperative. By gaining a deeper understanding of the impact of sleep on childhood obesity in the context of epigenetics, overall health outcomes may improve. This review primarily focuses on sleep and its impact on epigenetic modifications within various childhood obesity-related genes. Studies regarding sleep-related genes associated with obesity will be included. To identify pertinent human studies, a non-systematic search was conducted across electronic databases, including PubMed, Web of Science, and Medline, utilizing search terms such as epigenetics, obesity, childhood obesity, sleep, DNA methylation, gene expression, epigenetic modifications, sleep quality, sleep duration, and transgenerational epigenetic modifications.

## 2. Sleep and Childhood Obesity

Sleep is a fundamental aspect of a child’s overall health and well-being, playing a crucial role in physical, mental, and emotional development [34]. Despite its significant role in growth and development, sleep deficiency is becoming a major public health concern for children worldwide. Affecting a staggering 20% to 60% of children, sleep deficiency is a broad term encompassing insufficient sleep and disruptions in sleep quality [35,36,37,38]. This means children may not be getting enough sleep at night (sleep deprivation), have inconsistent sleep schedules, experience restless sleep, or have underlying sleep disorders that prevent them from getting the restorative sleep they need [35,36,37,38]. The impact of this sleep deficiency can be significant, affecting children’s physical and mental health, academic performance, and overall well-being. As we age, our sleep requirements evolve. School-aged children (ages 6–12), according to the Centers for Disease Control and Prevention (CDC), need a substantial amount of sleep, between 9 and 12 h each night [18]. This is significantly more than the typical adult recommendation of 7 or more hours [39]. This difference reflects the critical growth and development happening during these childhood years. The importance of sleep in relation to childhood obesity cannot be overstated, as growing evidence highlights the intricate connection between inadequate sleep and an increased risk of obesity in children. Insufficient sleep, meaning a lack of sleep needed to stay awake and alert during the day, can manifest in several ways [40]. It can include irregular sleep patterns like going to bed and waking up at inconsistent times, simply not getting enough sleep each night, experiencing restless or poor-quality sleep, or having a sleep disorder that disrupts regular sleep patterns [40]. This lack of quality sleep is increasingly recognized as a significant contributing factor to childhood obesity [40]. Sleep helps aid muscle recovery, promote growth and repair, facilitate memory consolidation and learning, sustain attention and concentration, and enhance immune system function, all of which are crucial for school-aged children [32]. Due to this intrinsic need for sleep, a lack thereof can lead to a broad spectrum of disruptions within the body, including an increased risk of obesity [35]. Therefore, as the rate of childhood obesity continues to rise and becomes a more pressing global issue, it is important to recognize sleep as a major contributor.

While there is ample evidence attesting to the relationship between sleep duration, sleep issues, and childhood obesity, there are few studies investigating the association between sleep timing and obesity in youth [41]. Inadequate sleep timing involves going to bed late and maintaining inconsistent patterns, including fluctuations across the week and disparities between weekday and weekend sleep schedules, reflecting a ‘shifted’ or ‘delayed’ sleep phase [42]. A recent study by Skjåkødegård et al. (2021) looked at how sleep duration, problems, and timing were related to obesity in children [41] The study found a link between later sleep times and unhealthy weight-related behaviors (obesogenic behaviors) in children, suggesting that later sleep timing could be a risk factor for obesity [41]. Children with severe obesity were more likely to have irregular sleep schedules throughout the week and also experience more sleep problems, supporting this connection [41]. The study also found that higher screen time, an obesogenic behavior, was found to be positively related to sleep problems [41]. This research suggests a connection between later sleep timing, obesogenic behaviors in children and adolescents, and a potential increased risk of obesity [41]. In another study by Miller et al. (2014) examining sleep timing and obesity in early childhood, it was discovered that a late bedtime (after 9 p.m.) intensified and autonomously forecasted the link between insufficient sleep duration and obesity [43]. In another study conducted by Golley et al. (2013) [44], research with school-age children and adolescents (aged 8–17 years) revealed that late bedtimes were linked to adiposity, irrespective of sleep duration [44,45]. Additionally, school-age children and adolescents (9–16 years) exhibiting a late bedtime/late wake time pattern were more prone to being overweight, engaging in higher screen time, and having lower physical activity levels compared to those with an early bedtime/early wake time pattern [46]. Recent research has shown that variations in sleep schedules between weekends and weekdays are connected to adiposity in school-age children and adolescents, regardless of sleep duration [47]. While these observations require validation in larger samples, it implies that a late bedtime is a distinct factor contributing to the risk of obesity in youth. Thus, these studies highlight the importance of considering other aspects of sleep when conducting research and clinical work related to obesogenic effects in youth [41].

The duration of sleep is a critical component of sleep itself that is essential for the optimal function of our bodies. Research has consistently shown that children who experience insufficient sleep are more likely to exhibit unhealthy weight gain and obesity [48]. Recent research has unveiled strong evidence for a negative association between sleep duration and overweight and obesity among primary school-aged children [49]. However, further insight on sleep quality, sleep efficacy, and sleep timing would benefit future research [49]. It is also important to consider how the relationship between sleep and obesity is multifaceted, involving complex physiological and behavioral mechanisms. For more than a decade, it has been evident that insufficient sleep duration could contribute to increased weight gain [50]. This phenomenon can be linked to the hormonal changes that help regulate appetite, specifically the secretion of leptin and ghrelin [50,51]. Although a broad epidemiological study indicated that chronically short sleep duration in school-age children was linked to lower leptin levels, a smaller experimental study manipulating sleep duration found higher leptin levels associated with short sleep [52]. While considerable research has concentrated on appetite-regulating hormones and other biologically mediated pathways concerning sleep and childhood obesity, another recent observation indicates a tendency to elevate energy intake following sleep restriction without accompanying hormonal changes [53,54]. This underscores the significance of behavioral or environmental factors, such as screen time and physical activity, and how these external factors can play a crucial role in pathways associated with eating behavior. Methodological variations between these studies may explain the divergent results; however, neither study considered epigenetics in the regulation of metabolism [55]. Hence, it is plausible that sleep timing and sleep duration collectively influence the physiological processes involving the epigenome relevant to weight gain [56].

Understanding how sleep patterns contribute to obesity risk beyond sleep duration across various developmental stages is crucial for comprehending longitudinal and mechanistic associations and devising effective interventions [48]. Developing a consistent and routine sleep pattern during early childhood could improve metabolic regulation and set the stage for healthier sleep in later years [48]. Sleep schedules experience notable changes as individuals grow, and the impact of sleep timing on obesity appears to emerge in childhood [57]. Studies involving adults have revealed distinct biological and behavioral pathways connecting sleep and obesity, with many of them revolving around the timing of sleep and eating behavior [48]. A recent meta-analysis by Liu et al. (2024) revealed mixed results on the effect of sleep interventions across studies on BMI, other weight-related outcomes, diet, physical activity, and sleep [58]. This study highlights the need for future studies with a rigorous randomized controlled trial (RCT) design that would incorporate objective measures of sleep to best cultivate guidelines and recommendations on sleep for overweight and obese youth [58]. Thus, to further understand the impact of sleep on the metabolic effects in youth, epigenetics must be taken into consideration.

## 3. Sleep and Epigenetics

Scientific understanding of how sleep influences the epigenome is still evolving, but there is strong evidence that sleep deprivation can trigger changes in epigenetic markers, impacting gene expression and overall health. Gene expression undergoes regulation through various processes, with epigenetic mechanisms playing a significant role. In a general context, epigenetics involves alterations to the genome that do not change the fundamental genetic sequence. Over recent years, multiple studies have investigated the link between sleep and the epigenome through epigenetic mechanisms, including DNA methylation, histone modification, and non-coding RNAs [59]. The DNA modifications resulting from these epigenetic mechanisms collectively constitute the epigenome, which exhibits variability across cells and generations due to the dynamic and environmentally sensitive nature of epigenetic modifications. To fully grasp the intent of this review, the epigenetic mechanisms regarding sleep and their potential impact on childhood obesity and beyond need to be further explained.

### 3.1. DNA Methylation and Sleep

Introducing a methyl group to a cytosine-guanine dinucleotide (CpG), a process referred to as DNA methylation, constitutes the most prevalent epigenetic modification [60]. The overall configurations of DNA methylation throughout the genome are commonly referred to as the methylome. When DNA methylation takes place within the promoter region to the initial exon of a gene, it generally leads to a decrease in gene expression [61]. This decline in gene expression is, at least in part, linked to the presence of methyl-CpG binding protein 2 (MeCP2). MeCP2 binds to the methylated DNA, instigates alterations in chromatin structure, and recruits transcriptional repressors to the methylation site [62,63]. The abundance of MeCP2 in the brain underscores the prevalence of functional DNA methylation, which is crucial for normal brain function [60]. Any disruptions to this process may result in neurobiological alterations, affecting sleep and other metabolic conditions, such as the development of obesity as early as childhood.

DNA methylation levels across the epigenome are established and managed by a group of enzymes known as DNA methyltransferases (DNMTs) [60]. This enzyme family comprises DNMT1, DNMT2, DNMT3A, DNMT3B, and DNMT3-like (DNMT3L) [60,64]. DNMT1 is involved in maintaining the methylome integrity during DNA replication, while DNMT3A and DNMT3B are responsible for de novo DNA methylation [65]. Despite lacking enzymatic activity, DNMT3L plays a crucial role in DNA methylation during development and is frequently considered a companion to DNMT3A and DNMT3B [64]. DNMT2’s function remains somewhat unclear, but it possesses the enzymatic capability to methylate DNA and is also recognized as an RNA methyltransferase [66,67]. In contrast to DNMTs, the ten-eleven translocation (TET) enzymes, including TET1, TET2, and TET3, govern DNA demethylation by introducing a hydroxyl group to the methyl group [68]. This process yields an intermediate functional base known as 5-hydroxymethylcytosine (5hmC), believed to be of particular significance in the brain and its concentration in genes associated with synapses, contributing to the risk of developing obesity in early childhood [69,70].

There is considerable evidence pointing to the significant impact of sleep on DNA methylation [60]. In a human study by Wong et al. (2015), twins with different inclinations for specific times of the day exhibited distinct DNA methylation patterns after analyzing buccal cell tissue [71]. Additionally, Huang et al. (2017) found that individuals classified as short sleepers exhibited changes in DNA methylation patterns in 52 genes compared to those identified as long sleepers after analyzing blood samples [72]. Furthermore, a body of evidence links the methylation status of circadian clock genes to lack of sleep across a range of tissues [73]. Qureshi et al. (2014) specifically examined the DNA methylation levels of circadian rhythm genes in males following one night of sleep deprivation, revealing significant hypermethylation in adipose tissue samples [73]. However, these methylation changes did not correspond to alterations in gene expression, suggesting a potential delayed response or the involvement of additional factors such as histone modifications and non-coding RNAs [73]. These investigations collectively provide compelling evidence that both short-term and prolonged sleep deprivation can lead to similar alterations in the epigenetic profile of circadian genes. However, more research is needed, especially in the context of youth and its relation to obesity.

Aside from the changes in DNA methylation identified in circadian genes, alterations in DNA methylation profiles have been documented in genes linked to metabolic functions [74]. Sleep deprivation commonly disrupts metabolic pathways, and metabolic enzymes play a crucial role in governing hippocampal memory affected by sleep deprivation [75]. Following sleep deprivation in male blood samples, a noticeable rise in DNA methylation was identified near the transcription start site of Stearoyl-CoA Desaturase 1 (SCD1), a pivotal enzyme in fatty acid desaturation [76]. Previous research emphasizes that sleep deprivation can induce alterations in DNA methylation, potentially disturbing essential physiological processes tied to metabolism and circadian rhythm regulation, thus increasing the risk of developing obesity across the lifespan [77].

A genome-wide study conducted by Möller-Levet et al. (2013) identified 269 probes significantly altered in sleep-deprived subjects. This finding aligns with previous research and suggests potential genes that undergo changes in both expression and DNA methylation due to shifts in sleep duration and timing [78]. These studies suggest that DNA methylation can negatively regulate gene expression and work with other epigenetic mechanisms to alter gene expression [79]. Further GWAS studies will uncover the relationship between sleep and obesity amongst various age groups.

To delve further into genome-wide methylation levels in response to sleep deprivation, Massart et al. (2014) measured alterations in 5hmC patterns in 4697 genes due to sleep deprivation, primarily affecting exons and the 3′ untranslated region (UTR) [77]. Elevated expression of Dnmt3a1 and Dnmt3a2 genes following sleep deprivation correlated with hypermethylation at the 3′UTR, underscoring the connection between hypermethylation and DNA methylation [77,80]. While alterations in DNA methylation are evident in specific genomic regions and throughout the genome in response to sleep deprivation, studies specific to children are limited [60]. Despite this limitation, these changes are associated with modified gene expression and contribute to the identification of pathways dysregulated by loss of sleep [60]. Further research is needed to characterize DNA methylation patterns specifically related to sleep, pinpoint target genes influenced by sleep deprivation, and understand the implications of these changes.

### 3.2. Histone Modifications and Sleep

Histone modifications play a pivotal role in the field of epigenetics. This process entails chemical changes to histone proteins, the structural elements around which DNA is coiled in the cell nucleus. Importantly, these alterations bring about a shift in gene expression without changing the underlying DNA sequence [59]. Histone proteins contribute to packaging and organizing DNA into a condensed structure known as chromatin [3]. The configuration of chromatin, a key determinant of gene expression, can undergo chemical modifications through processes such as methylation, acetylation, phosphorylation, and more, primarily occurring on the histone tail extending from the chromatin [59]. These modifications influence the degree of DNA wrapping around histones, thereby affecting both gene accessibility and expression.

The extensively studied post-translational modification (PTM) known as histone acetylation involves lysine acetyltransferases (HATs/KATs), which transfer an acetyl group from acetyl-CoA to lysine residues on a target protein [81]. Conversely, lysine deacetylases (HDACs/KDACs) facilitate the reverse reaction [81]. The relationship between HATs and HDACs plays a pivotal role in regulating gene expression events crucial for neuronal function, encompassing behavior and memory [60,81]. Histone acetylation diminishes interactions between histones and DNA, resulting in a more open chromatin conformation that promotes the recruitment of transcriptional machinery [59]. Within the chromatin, HATs showcase discernible substrate preferences, designating histones for specific events [60]. The patterns of histone acetylation, when coupled with other post-translational modifications, form a regulatory code that determines signals for gene expression [60]. Recent discoveries propose a connection between metabolic enzymes, neuronal histone acetylation, and the process of memory formation [76]. As sleep deprivation has a significant impact on gene expression, it is conceivable that the lack of sleep induces changes in histone modifications, thereby affecting gene transcription [60]. This potential disruption in gene regulation due to sleep disturbances might contribute to the development of chronic conditions like childhood obesity, highlighting the importance of adequate sleep for overall health. 

The circadian clock is intricately connected to the chromatin’s epigenetic state, where a close interplay between HATs and HDACs tightly governs the transcriptional rhythm of clock-target genes [82,83]. The CLOCK protein, functioning as both a transcription factor and histone acetyltransferase, directs acetylation on histones H3 and H4, serving as a crucial regulator for genes linked to circadian rhythm [84,85]. In the context of childhood obesity, disrupted sleep has been linked to the disturbance of major circadian genes, such as CLOCK, BMAL1, CRY1, and PER1, impacting hormone regulation, metabolism, and energy expenditure, potentially contributing to weight gain in early development [86]. While these circadian genes may directly influence fat storage or appetite regulation, additional research is needed to understand the epigenetic mechanisms modulating the circadian genes crucial for maintaining circadian rhythm, sleep homeostasis, and a healthy metabolic state [84,85,86,87]. By identifying specific histone modifications that that undergo changes after sleep deprivation, advancements in finding potential therapeutic targets for addressing sleep-related disorders and sleep deprivation are made possible [84,85,86,87]. Therefore, further studies regarding epigenetic markers are warranted, particularly to elucidate how these disruptions contribute to the development of childhood obesity through altered circadian rhythmicity and metabolic processes.

### 3.3. Sleep-Related Non-Coding RNAs

Various types of non-coding RNAs exist, although only a few have been explored in the context of sleep [60]. These RNAs can be classified by size, with long non-coding RNAs (lncRNAs) and microRNAs being the most prevalent [60]. While much about their functions remains unknown, their presence in both healthy and abnormal cells suggest a probable role in various biological pathways, including those related to sleep and metabolism [60]. 

LncRNAs exceeding 200 base pairs perform diverse roles, including recruiting epigenetic and regulatory components to specific genomic sites and regulating processes such as splicing and translation [88]. When located in the cellular nucleus, they alter chromatin structure, engage with enzymes responsible for chromatin modifications, and consequently impact gene expression [89]. Understanding the impact of non-coding RNAs, especially lncRNAs associated with circadian rhythms, is crucial. The deletion of lncRNA 116HG, for example, disrupts circadian genes such as Clock, Cry1, and Per2, contributing to sleep gene dysfunction and hormonal/metabolic changes linked with genes related to sleep and obesity [90]. LncRNAs are also directly involved in fat storage, energy expenditure, and appetite regulation as well [90]. Despite existing correlations, further research is necessary to establish a clear cause-and-effect relationship between lncRNA disruption and genes associated with sleep regulation and adiposity. 

Insufficient sleep levels caused by work- or lifestyle-related activities, health conditions, or environmental stressors can all lead to subsequent changes in DNA methylation, histone modifications, and non-coding RNAs [59]. These mechanisms can act independently or collaboratively to modify the expression of target genes and proteins [59]. The utilization of DNA-sequencing analyses will aid in identifying these epigenetic biomarkers associated with both sleep and obesity, providing a deeper understanding of the epigenetic modifications resulting from changes in sleep timings and durations and their potential contribution to the rising incidences of obesity in both children and adults.

## 4. Linking Sleep and Childhood Obesity from an Epigenetic Standpoint

Childhood obesity is a complex issue with roots in both genes and environment. Increasing evidence in epigenetics suggests that genetic predispositions alone do not exclusively dictate the onset of heritable chronic diseases; instead, they may be altered—either mitigated or intensified—by environmental influences. The exciting news is that epigenetics suggests that genes are malleable when it comes to obesity risk during childhood. Even if a child has a genetic predisposition, a healthy lifestyle can help. Getting 9–12 h of high-quality sleep at home is one important factor [18]. By prioritizing healthy habits, we can reduce the epigenetic risk associated with genes linked to childhood obesity. Therefore, the choices we make, such as ensuring a child gets enough sleep, can influence how genes are expressed, thereby lowering the risk of developing obesity. This knowledge provides the ability for individuals to take control and create a healthier environment for children to grow up in.

Recent progress in understanding the epigenetic underpinnings of sleep traits and sleep disorders brings hope for unraveling the complex connection between sleep and childhood obesity [33]. In a meta-analysis of epigenome-wide association study findings conducted by Sammallahti et al. (2022), DNA methylation was assessed in cord blood at birth across 11 cohorts and in peripheral blood in children aged 4–13 years from eight cohorts [91]. The study examined outcomes such as parent-reported sleep duration, sleep initiation, and fragmentation issues, as well as measures including sleep duration, sleep onset latency, and wake-after-sleep-onset duration. However, no significant findings were reported [91]. In a systemic review by Leader et al. (2021) focusing on the epigenetics of obstructive sleep apnea syndrome (OSA), human patients with OSA had unique epigenetic changes—compared to healthy control patients—which were also commonly identified in genes associated with metabolic and inflammatory pathways [92]. While the available studies are limited, research such as this provides valuable insight for the development of epigenetic markers for the diagnosis and treatment of OSA and other sleep-related conditions [92]. Through further epigenome-wide investigations, robust biomarkers for the identification of sleep disorders or the impact of the loss of sleep can be identified among both children and adults [92]. Therefore, exploring the interaction between sleep loss, patterns of sleep timing, sleep disorders, and their independent contributions to epigenetic changes that influence the risk of obesity warrants further development.

**Figure 1:**
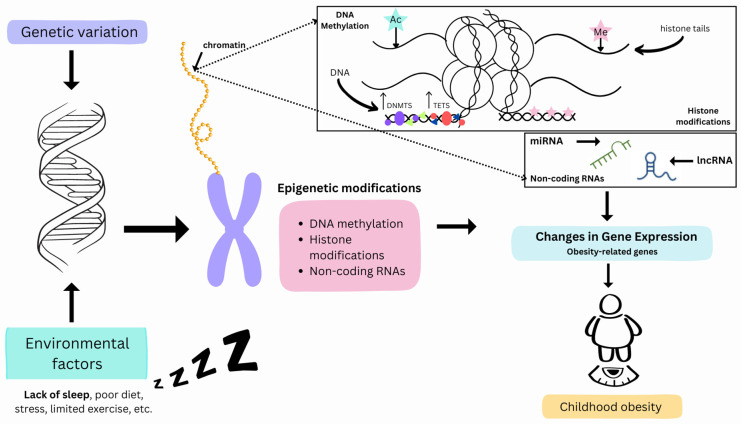
A schematic representation of the multifaceted interplay between genetic predisposition, environmental stressors, and key epigenetic modifications in the context of childhood obesity. The depicted arrows illustrate the interconnectedness of these factors. The purple and red dots symbolize DNA methyltransferase (DNMT) and ten-eleven translocation (TET) enzymes, respectively. Green triangles represent methylated cytosines, while blue triangles represent unmethylated cytosines. The blue and pink stars represent histone acetylation (Ac) and methylation (Me) modifications on histone tails.

In a recent cross-sectional genome-wide analysis by Lahtinen et al. (2019) examining DNA methylation in relation to insufficient sleep in individuals stemming from shift work disorder, the study revealed that lack of sleep was associated with loss of DNA methylation [93]. These findings reveal how DNA methylation patterns associated with sleep loss affect the modification of processes related to neurodegeneration, thus having a negative impact on metabolic effects [93]. Although this study focused on adults, it provides valuable insight into the epigenetics of sleep that can guide future research exploring the connection between sleep and childhood obesity [93]. Therefore, from this study, epigenetic modifications triggered by lack of sleep can be further investigated in relation to obesogenic effects. While inherent genetic variations can influence susceptibility, various environmental factors such as dietary patterns, sleep hygiene, and even toxin exposure demonstrably impact obesity risk without altering the underlying DNA sequence. These environmental cues trigger epigenetic modifications, manifested as DNA methylation, histone acetylation, and non-coding RNA involvement (Figure 1). These modifications act as molecular switches, regulating gene expression and potentially increasing or decreasing a child’s obesity risk. Notably, even with a genetic predisposition, a healthy lifestyle emphasizing proper sleep and a balanced diet can potentially counteract negative epigenetic effects, ultimately mitigating the risk of childhood obesity.

Another critical aspect to consider with the epigenetic impact of sleep on the body is timing [32]. Although the circadian clock plays a role in both sleep and metabolism, only a handful of shared genes, such as FTO, have surfaced in epigenome-wide association studies (EWASs) of sleep and childhood obesity [32]. Despite these challenges, alterations to the epigenome have been observed following acute sleep deprivation, although individual variations exist [32]. Research investigating the influence of sleep deprivation on the epigenome has been conducted using both animal and human model systems. However, it is crucial to acknowledge the limitations inherent in both systems when interpreting the study findings. Human studies on sleep deprivation often rely on accessible samples, primarily saliva or blood, and may be significantly influenced by environmental stressors [32]. Moreover, studies involving acute sleep deprivation may not fully replicate the continuous chronic wake time commonly experienced by humans [32]. In the future, these epigenetic modifications could potentially serve as indicators of sleep loss or be targeted for therapeutic strategies in addressing sleep-related disorders. This approach, in turn, could help mitigate the risk of developing obesity in youth, positively impacting today’s society. Performing repeated evaluations of methylation in conjunction with concurrent assessments of sleep may provide insights into the patterns of DNA methylation changes over time and their enduring effects on sleep [92]. Additionally, delving into alternative epigenetic mechanisms, such as histone modifications or tissue-specific effects, could be valuable, particularly if large-scale analyses of these mechanisms become feasible for future research endeavors [92]. Therefore, a comprehensive exploration of various epigenetic mechanisms, coupled with a nuanced understanding of their interplay with sleep patterns, holds the potential to deepen our knowledge of the intricate relationship between the epigenome and sleep. This multifaceted approach may pave the way for targeted interventions and therapeutic strategies aimed at promoting healthier sleep and mitigating the long-term consequences of disrupted epigenetic regulation. Therefore, additional research is warranted to unveil the longitudinal connections between epigenetic modifications and children’s sleep.

## 5. Transgenerational Epigenetic Changes and Changes in Sleep Patterns

In the past decade, the overall sleep duration among the population has decreased. Parents’ experiences may influence their children’s health through epigenetic modifications on their genes, which are chemical markers that can be passed down to future generations, acting as a type of inherited trait. The “fetal origins of adult disease” (FOAD) proposed by David Barker, highlights the vulnerability of fetuses and infants to adverse intrauterine and early developmental environments. These exposures can increase the susceptibility of adult offspring to various metabolic diseases [94]. As women today take on more prominent roles in the workforce without significantly reducing their domestic responsibilities, the priority given to sleep often diminishes in women’s daily schedules. From an epigenetic perspective, this societal shift has an impact on future generations, as the alteration in maternal sleep patterns and duration affects the epigenome and overall health of a child.

Insufficient sleep stands as a pivotal risk factor for the onset of metabolic, cardiovascular, neuropsychiatric, immunologic, and gastrointestinal issues, as well as a compromised prognosis for cancer in the later stages of life [59]. Additionally, sleep disturbances during pregnancy can lead to negative outcomes for both the mother and the child [95]. Pregnancy is a dynamic period marked by significant lifestyle changes. Numerous guidelines exist for maintaining maternal health through diet, exercise, and sleep [96]. However, sleep disorders, including fragmented or inadequate sleep, are prevalent among pregnant women, especially during late gestation [97,98,99,100]. Growing evidence suggests a potential link between maternal sleep and offspring health through epigenetic modifications [101]. In a study by Carroll et al. (2023) looking at insufficient sleep and its link to accelerated biological aging in adults, short sleep in postpartum at 6 months following a birth was predictive of older 12-month Intrinsic Epigenetic Age Acceleration (IEAA) [102]. These findings suggest that insufficient sleep duration during the early postpartum period is associated with accelerated biological aging; however, additional research with a larger sample size is warranted to replicate these results [102]. The likelihood of developing a chronic disease, such as obesity, can be heightened with an accelerated biological age; therefore, it is imperative to further investigate the impact of the intrauterine environment on the epigenome [95]. More recent research supports the association between gestational sleep deprivation and offspring health. A study by Harskamp-van Ginkel et al. (2020) using data from two European cohorts with diverse ethnic and demographic characteristics found that gestational sleep deprivation might be linked to an increased risk of overweight and higher blood pressure in offspring up to 11 years old [103]. Interestingly, this study also showed a stronger association in girls compared to boys [103]. These findings underscore the need for further research on the impact of sleep quality during pregnancy, particularly its potential sex-specific effects. Additionally, developing official sleep recommendations for pregnant women is crucial. Based on this study, avoiding sleep deprivation (defined as less than 6 h of sleep) throughout pregnancy seems prudent [103]. A recent study by Briollais et al. (2021) provided significant evidence for the role of breastfeeding in modulating epigenetic factors as a possible mechanism translating its benefits on child development [104]. This study suggests that exclusive breastfeeding during infancy elicited more significant DNA methylation variations during infancy compared to other childhood growth periods [104]. At the genome-wide level, 13 CpG sites in girls and 2 CpG sites in boys were identified that mediated the association between exclusive breastfeeding and longitudinal BMI [104]. Furthermore, enrichment of CpG sites within microRNAs and key pathways, including AMPK signaling, insulin signaling, and endocytosis, strengthens this link [104]. These findings support the concept of the early postnatal period as a pivotal developmental window for substantial DNA methylation modifications, potentially mitigating the development of overweight and obesity from infancy to early childhood [104]. Since rapid growth during these early stages has been associated with increased risk of later-life obesity, exclusive breastfeeding might play a crucial role in preventing childhood and adult overweight/obesity through early-life DNA methylation mechanisms [104]. In relation to sleep, a study by Jafar et al. (2021) revealed how, despite more night awakenings, fully breastfed infants have overall longer night- and total-sleep durations than formula-fed infants [105]. Thus, the introduction of breastfeeding can be attributed to genes associated with sleep and obesity in early childhood [105].

Building upon prior research, Meng et al. (2022) embarked to elucidate the association between three distinct maternal sleep dimensions during late pregnancy and offspring adiposity markers [106]. Their investigation further aimed to explore the potential mediating effect of cord blood DNA methylation [98]. Employing an epigenome-wide association study design, they sought to identify maternal sleep-related CpG sites potentially linked to maternal sleep patterns [106]. Notably, the study revealed a significant association between later maternal sleep timing in late pregnancy and increased childhood adiposity in offspring, with cord blood DNA methylation emerging as a putative mediator in this relationship [106]. This groundbreaking research offers the first human-based evidence for cord blood methylation acting as a mediator between the midpoint of maternal sleep during late pregnancy and the child’s adiposity status [106]. Furthermore, this demonstrates how alterations arising from later maternal sleep timing could serve as a mechanism for the fetal programming of adiposity [106]. However, future studies will require larger cohorts to solidify these findings.

Understanding the factors that contribute to childhood obesity are crucial, particularly the distinction between inherited and non-inherited forms. Traditional genetics focuses on DNA sequence variations passed down from parents, explaining a portion of obesity risk. However, epigenetics offers a new layer of complexity. Epigenetic modifications can influence gene expression without altering the DNA sequence itself. These chemical tags can be influenced by environmental factors like sleep and passed down to future generations. This makes epigenetics a unique player, potentially explaining how a parent’s environment and lifestyle choices might influence their child’s obesity risk through these transgenerational epigenetic marks, even if there is no change in the underlying DNA sequence. This stands in contrast to non-hereditary obesity, which is primarily driven by factors like excessive calorie intake and lack of physical activity that directly impact an individual’s weight. Despite the ability of epigenetic modifications to be transmitted across generations and persist, these changes exhibit a degree of reversibility [96]. By understanding the distinct molecular signatures of epigenetics, obesogenic genes, and non-hereditary factors, we can develop more targeted strategies for preventing and managing obesity across generations. Also, by further investigating the impact of sleep and other lifestyle choices on the maternal and child epigenome, we can develop strategies to promote healthy lifestyle habits across generations as well. Prioritizing sleep for mothers and supporting breastfeeding practices are crucial steps in this fight. Further research is needed to solidify these findings, but the message is clear—promoting healthy habits during critical developmental windows can potentially mitigate the risk of chronic diseases like obesity in future generations.

## 6. Limitations and Future Direction

Several limitations were identified in the reviewed studies. To start, there was variability in methods employed for analyzing CpG methylation for both sleep- and obesity-related genes investigated. Additionally, the research considered in this review involved participants from diverse groups, introducing variability in estimating methylation linked to population-specific effect magnitudes. Difficulty finding ample studies involving children was also a limitation, as most sleep-methylation studies focused on adult populations. Moreover, variations in sleep habits resulting from diverse sleep disorders, disruptions, and patterns further complicated the analysis in this review. To further understand, more studies devoted to the relationship between mother and child in relation to sleep and the onset of obesity are warranted. Such insight would elucidate the influence of sleep on epigenetic modifications, paving the way for affordable and accessible therapeutic strategies to enhance overall health, starting in the womb. Also, to enhance the understanding of DNA methylation and its role in gene regulation, in the context of childhood obesity, more intervention studies would prove useful. These studies should encompass control groups and involve both mother and baby for a more comprehensive understanding from an epigenetic standpoint. 

The mechanisms underlying the reversal of epigenetic modifications in human obesity remain inadequately understood. Incorporating healthy lifestyle practices, including eating a balanced diet, participating in regular exercise, and attaining sufficient sleep, holds promise for alleviating stress and fostering epigenetic modifications, as well as making a positive impact on the obesity-related epigenome. Despite this, there is a conspicuous gap in today’s research, with limited exploration into whether alterations in sleep patterns and environmental factors can indeed facilitate the reversal of these epigenetic modifications specific to childhood obesity. Therefore, a more in-depth investigation into this research area is essential.

Overall, the current research findings underscore the importance of incorporating a multifaceted approach to sleep analysis in childhood obesity research and clinical practice, moving beyond mere sleep duration to encompass the chronobiological aspects of sleep. Future longitudinal and intervention studies, employing objective measures of sleep, are necessary to better understand the associations between sleep timing, sleep duration, sleep problems, and childhood obesity, and to explore the underlying epigenetic mechanisms of the sleep–obesity link. 

## 7. Conclusions

This comprehensive review explores the influence of sleep on the epigenetic modification of diverse obesity-related genes. Current literature proposes strong evidence for a negative association between sleep and obesity among youth, but delving into the epigenetic mechanisms involved has only begun. The disruptions in sleep that induce various changes in methylation at various CpG sites influence the regulation and modulation of certain genes. This, in turn, impacts several metabolic pathways, thus increasing the risk of obesity at a young age. However, varying tactics or differing areas of sleep limit the conclusions that can be drawn from studies. To better understand the epigenetic impact of sleep timing, sleep duration, sleep quality, and sleep efficacy on the sleep–obesity association, future studies would benefit from implementing a combined approach to investigating these dimensions both simultaneously and longitudinally. By doing so, the influence of the multiple dimensions of sleep on the weight status of children can be assessed over time and as sleep requirements change. Furthermore, elucidating the importance of sleep on epigenetic modifications helps to promote the exploration of alternative lifestyle-based therapeutic approaches and develop attainable tools for achieving better sleep to improve overall health, starting in youth.

## Abbreviations

AMPK, activated protein kinase pathway; CBP, CREB-binding protein; CPG, cytosine-phosphate-guanine; CREB, cAMP response element-binding protein; DNMTs, DNA methyltransferases; EWAS, epigenome-wide association studies; FDR, false discovery rate; FOAD, fetal origins of adult disease; HATs/KATs, histone acetyltransferases; HDACs/KDACs, histone deacetylases; IEAA, intrinsic epigenetic age acceleration; lncRNAs, long non-coding RNAs; MeCP2, methyl-CpG-binding protein 2; OSA, obstructive sleep apnea syndrome; PTM, post-translational modification; REM, rapid eye movement; SCDI, stearoyl-CoA desaturase 1; TET1, ten-eleven translocation enzyme 1; TET2, ten-eleven translocation enzyme 2; TET3, ten-eleven translocation enzyme 3; T2DM, type 2 diabetes mellitus; UTR, untranslated region; WHO, World Health Organization; 5hmC, 5-hydroxymethylcytosine.
